# Incorrectly fitted footwear, foot pain and foot disorders: a systematic search and narrative review of the literature

**DOI:** 10.1186/s13047-018-0284-z

**Published:** 2018-07-28

**Authors:** Andrew K. Buldt, Hylton B. Menz

**Affiliations:** 1 La Trobe Sport and Exercise Medicine Research Centre, School of Allied Health, La Trobe University, Bundoora, Melbourne, VIC 3086 Australia; 2 Discipline of Podiatry, School of Allied Health, La Trobe University, Melbourne, VIC 3086 Australia

## Abstract

**Background:**

Correct footwear fitting is acknowledged as being vitally important, as incorrectly fitted footwear has been linked to foot pathology. The aim of this narrative review was to determine the prevalence of incorrectly fitted footwear and to examine the association between incorrectly fitted footwear, foot pain and foot disorders.

**Methods:**

A database search of Ovid MEDLINE and CINAHL yielded 1,681 citations for title and abstract review. Eighteen articles were included. Findings were summarised under the categories of (i) children, (ii) adults, (ii) older people, (iii) people with diabetes and (iii) occupation- or activity-specific footwear. Differences in footwear fitting between sexes were also explored.

**Results:**

Between 63 and 72% of participants were wearing shoes that did not accommodate either width or length dimensions of their feet. There was also evidence that incorrect footwear fitting was associated with foot pain and foot disorders such as lesser toe deformity, corns and calluses. Specific participant groups, such as children with Down syndrome and older people and people with diabetes were more likely to wear shoes that were too narrow (between 46 and 81%).

**Conclusion:**

A large proportion of the population wear incorrectly sized footwear, which is associated with foot pain and foot disorders. Greater emphasis should be placed on both footwear fitting education and the provision of an appropriately large selection of shoes that can accommodate the variation in foot morphology among the population, particularly in relation to foot width.

## Background

Footwear has been used by humans for approximately 30,000 years [1]. Although originally worn as a protective covering for the foot, modern footwear is designed to fulfil a range of purposes, the accomplishment of which is judged by three criteria: form, function and fit [2]. *Form* relates to the aesthetic appeal of footwear, while *function* relates to the ability of footwear to accomplish its intended purpose, eg. to protect the feet of individuals who undertake activities that may present a risk of injury. Finally, *fit* pertains to how footwear can accommodate the morphology of the foot [3].

Footwear fitting is acknowledged as being vitally important as in most cases *fit* governs *function* [3]. This means that footwear cannot fulfil its intended purpose if it does not fit the foot correctly [2]. Furthermore, it has been suggested that incorrectly fitted footwear is a major contributor to the development of structural foot disorders, such as hallux valgus and lesser toe deformity [4, 5], as well as skin lesions, such as corns and calluses [6].

Correct footwear fitting is an inherently complex undertaking for two main reasons. Firstly, the footwear industry is currently unable to design and manufacture footwear that can conform to the three-dimensional morphology of all feet in the population [7, 8]. This is because foot morphology is highly variable between individuals, and there is limited variety in the shape of lasts used to construct footwear [9–12]. Secondly, footwear selection is not purely based on quantitative measurements of footwear shape and size, but may be influenced by qualitative factors [13, 14]. It is therefore probable that a substantial proportion of the population are wearing incorrectly fitted footwear. With this in mind, the aim of this review is to determine the prevalence of incorrectly fitted footwear and to examine the association between incorrectly fitted footwear, foot pain and foot disorders.

## Methods

An electronic database search was conducted in March 2018, using the online databases Ovid MEDLINE (1946-present) and CINAHL (1980-present). A set of search terms were derived, and to broaden the search, some terms were truncated with wildcard symbols. The following keywords search terms were used: foot OR shoe* OR footwear AND size* OR fit* OR length OR width OR ‘footwear fit*’ OR ‘shoe fit*‘AND pain* OR disorder* OR ‘foot pain’. Results were limited to human studies published in peer-reviewed journals. The electronic database search was supplemented by cross-checking citations and reference lists from relevant published studies.

A single reviewer (AKB) assessed all studies that were yielded from the search by title and abstract. Studies that fulfilled one or both of the following two criteria were included: (i) studies reported the prevalence of participants wearing incorrectly fitted footwear, and (ii) studies reported the association between wearing incorrectly sized footwear and foot pain or foot disorders.

## Results

### Search results

The search strategy yielded 1681 citations. Following title and abstract review, 18 articles were included and a narrative summary of the findings is provided. A summary of all included studies including participant characteristics, method of analysis and main findings are presented in Table 1.

**Table 1:** Summary of included studies

Authors	Participants	Method of analysis	Main findings
Akhtar et al. [32]	100 participants. 50 participants in foot pathology group, 12 men, 38 women, mean age: 49 (range 19–68) yrs. 50 participants in control group, 19 men, 31 women, mean age: 41 (range 19–65) yrs.	Foot length and width and footwear length and width was measured (method not mentioned). Incorrect sized footwear defined as difference greater than half a shoe size between foot and footwear.	• In the foot pathology group, 21 (45%) participants were wearing footwear at least half a size too small • 7 (14%) participants were wearing footwear half a size longer than their foot • 32 (64%) were wearing footwear narrower than their foot, mean 6 (range 2–9 mm) • In the control group, 7 (14%) participants were wearing footwear at least half a size too small • 13 (26%) participants were wearing half a size longer than their foot • 15 (30%) were wearing footwear that were narrower than their feet, mean 4 mm (range: 2–7 mm)
Burns et al. [18]	65 participants, 26 men 39 women, median age: 82 (range 64–93) yrs. Participants were consecutive admission to hospital rehabilitation unit.	Foot length was measured with the participant in a sitting position using a standard ‘Clarks’ measuring stick. Foot width was measured with calipers across the widest part of the metatarsal heads Footwear was measured according to shoe size and dimensions measured with calipers. Incorrect sized footwear defined as difference between foot and footwear greater than half a standard British shoe (7 mm) for length or one size for width.	• 32 (49.2%) participants wore footwear that was too long • 3 (4.6%) were wearing footwear that was too short • 21 (32%) were wearing footwear that was too wide • 2 (3%) were wearing footwear that was too narrow • 47 (72%) of participants wore footwear that was incorrectly fitting based on either width or length • 42 (65%) of participants wore footwear that was too big (too long, too wide or both) • 4 (6%) or participants wore footwear that was too small (too short, too narrow or both) • Incorrect shoe length was significantly associated with increased ulceration • Foot pain was significantly associated with incorrect footwear length
Carter et al. [26]	101 participants, 51 men, 51 women, mean age: 52 ± 14.5 yrs. All participants diagnosed with inflammatory arthritis.	Fit assessed using previously published footwear assessment tool. Appropriateness of shoe size determined according to length, width and depth.	• 69 (68.3%) participants wore incorrectly fitted shoes • 62 (61.3%) participants wore shoes that were too short • 39 (38.6%) participants wore shoes that were too narrow • 31 (30.6%) participants wore shoes that were too shallow
Chaiwanichsiri et al. [19]	213 participants, 108 men, 105 women, mean age: 68.7 ± 5.4 yrs. Mean BMI: 24.7 ± 3.3. All of participants were ethnically Thai.	Foot length, width, arch length, toe depth and heel width were measured with the participant in a sitting position using the Chula foot calliper. Internal footwear dimensions were measured using Chula shoe calliper and tape measure. Incorrect sized footwear defined as at least 5 mm difference between the foot and footwear for length, width, toe box measurements.	• 50% of women and 34.3% of men were wearing footwear that was narrower than their foot by greater than 5 mm • 22% of participants (35.5% of women) who were wearing footwear that were smaller than their feet reported foot pain compared to 9.5% of participants who were wearing appropriately sized footwear
De Castro et al. [28]	399 participants, 172 men, 227 women, mean age: 69.6 ± 6.8 yrs. 34 women and 38 men reported having diabetes.	Foot length was measured during relaxed standing with a calliper (distance between the most prominent point in the calcaneal tuberosity region and the 2nd toe). The participant reported footwear size. Incorrect sized footwear defined as foot length at least 2 mm difference between foot length and reported footwear size dimensions.	• 110 women (48.5%) and 119 men (69.2%) wore incorrect size footwear (> 2 mm difference) based on foot and shoe length • 29 women (12.8%) and 57 men (31.1%) were wearing footwear at least 1 cm longer than their feet • 1 (0.6%) man was wearing shoes shorter than his feet • There was a significant association between men and wearing incorrectly sized footwear • 19 women or 55.9% of all women with diabetes and 31 or 81.6% of all men with diabetes were wearing incorrectly sized footwear • Incorrectly sized footwear was associated with ankle pain in women
Dobson et al. [24]	270 participants, all men, mean age 38.3 ± 9.8 yrs., height 178.9 ± 5.7 cm, weight 93.2 ± 12.5 kg. All participants were underground coal miners. Participants who wore sizes 9, 10, 11 or 12 were selected for analysis.	Three-dimensional foot scans during bipedal stance of participants’ feet were taken. Dimensions of footwear worn by participant (either lace up boots or gum boots) were measured by scanning footwear plaster moulds in the same manner as participants’ feet. Moulds were created by pouring plaster of Paris into footwear. Foot and footwear dimensions were compared.	• There was a significant difference between 3-dimennsional foot dimensions and 3-dimensional footwear dimensions • Participants were wearing footwear that were substantially longer than their feet • Width of the forefoot and heel areas of footwear were not wide enough for participants’ feet
Frey et al. [23]	356 women, average age 42 yrs. Participants had no history of diabetes, rheumatoid arthritis, previous foot trauma or surgery. Any foot deformity was recorded.	Foot tracings were taken during relaxed weightbearing. Foot width was defined as the widest line perpendicular to a longitudinal bisection of the foot. The shoe was traced and shoe width was defined as the widest line perpendicular to a longitudinal bisection. Foot width and shoe width were compared.	• 88% of participants were wearing footwear that was narrower than their foot (average 1.2 cm) • In participants who had no foot pain, the average foot–footwear width discrepancy was 0.56 cm (20% of all participants) • For participants without deformity the average foot length–footwear width discrepancy was 0.60 cm (23% of all participants) • Of participants with a foot narrower than or equal to footwear, 64% had foot pain and 57% had foot deformity • Of participants with foot wider than footwear, 84% had foot pain and 79% had foot deformity
Frey et al. [22]	255 women, average age 41 yrs. Participants had no history of diabetes, rheumatoid arthritis, previous foot trauma or surgery.	Foot tracings were taken during relaxed weightbearing. Foot width was defined as the widest line perpendicular to a longitudinal bisection of the foot. The shoe was traced and shoe width was defined as the widest line perpendicular to a longitudinal bisection. Foot width and shoe width were compared.	• 86% of participants were wearing footwear that were narrower than their feet (average 0.88 cm) • In participants who had no foot pain the average foot width-footwear discrepancy was 0.58 cm • In participants without deformity, the average forefoot width-footwear width discrepancy was 0.52 cm
Harrison et al. [29]	100 participants, 52 men, 48 women, mean age 62.0 ± 14.9 yrs. All participant were diagnosed with diabetes, 36% of participants were administering insulin. The median length of time that participants had diabetes was 5.0 years.	Foot length was measured during standing with a ‘Clarks’ measurement device. Foot width was measured using sliding calipers. Footwear length was recorded using a measuring stick. Footwear width was measured using sliding calipers. Footwear length and width was subtracted from foot length and width. Incorrect sized footwear was defined as greater or less than half a US shoe size difference between footwear and foot length or greater or less than 0.7 cm difference between footwear and foot width.	• For the right foot, 63 (63%) of participants were wearing incorrectly sized footwear • 23 (23%) participants were wearing footwear that was too long • 10 (10%) participants were wearing footwear that was too short • 43 (43%) participants were wearing footwear that was too narrow • 1 (1%) participant was wearing footwear that was too wide • 29 (29%) participants were wearing footwear that was correct length but too narrow • For the left foot, 65 (65%) of participants were wearing incorrectly sized footwear • 24 (24%) participants were wearing footwear that was too long • 10 (10%) participants were wearing footwear that was too short • 46 (46%) participants were wearing footwear that was too narrow • 1 (1%) participant was wearing footwear that was too wide • 30 (30%) participants were wearing footwear that was correct length but too narrow • There was no association between incorrectly fitted footwear and neuropathy or absent pulses
Kusumoto et al. [21]	51 women, average age 21.3 years. All participants were Japanese students.	Foot length was measured during relaxed bipedal stance with spreading callipers from the centre of the posterior heel to end of longest toe. Sizes of leather footwear and sneakers were recorded. Foot length and length corresponding to footwear size compared.	• For leather footwear, 8% (right feet) and 2% (left feet) of participants wore same foot and footwear length • 73% (right), 75% (left) participants wore footwear longer than the foot (maximum 14 mm) • 20% (both left and right) wore footwear shorter than the foot (maximum 4 mm) • For sneakers 8% (right), 6% (left) of participants wore same foot and shoe length • 73% (right) 71% (left) of participants wore shoe longer than the foot (maximum 14 mm) • 18% (right) and 22% (left) of participants wore footwear shorter than the foot (maximum 6 mm)
Lim et al. [16]	50 participants, 28 men, 22 women, 10.6 ± 3.9 yrs., height 131.9 ± 18.6 cm, weight 39.6 ± 18.4 kg. All genetic variants of Down’s syndrome was present among participants.	Outline of each participants’ foot was traced onto a footprint mat while standing in relaxed bipedal stance. Maximum length and width of the participants’ foot and footwear was documented in millimetres. The outline of the sole of footwear was traced onto graph paper. Percentage difference between foot and footwear dimensions was calculated for length and width measurements.	• 29 (58%) participants wore footwear narrower than their feet • 5 (10%) participants wore footwear shorter than their feet • There was no significant association between foot structure and footwear fit
López-López et al. [27]	73 participants, 25 men, 48 women, 81.4 ± 6.4 yrs., height 162.9 ± 9.8 cm, weight 66.2 ± 12.2 kg. All participants were diagnosed with Alzheimer’s disease.	Foot length (distance between the posterior heel and the end of the longest toe) and width was measured during relaxed standing with a Brannock® device. Footwear length and width was measured with a Brannock® device. Definition of incorrect sized footwear not stated.	• 51 (69.9%) participants wore incorrect sized footwear • 28 (38.3%) participants wore footwear that was too long • 42 (57.5%) participants wore footwear that was too narrow • 22 (30.1%) participants wore footwear that was simultaneously too long and too narrow • 20 (27%) of participants wore footwear that was simultaneously the correct length but too narrow
López-López et al. [17]	62 participants, 29 men, 33 women, mean age 75.3 ± 7.9 yrs., height 164.1 ± 7.6 cm, weight 73.9 ± 11.3 kg. 31 participants in incorrectly fitted footwear group, 41 participants in correctly fitted footwear group.	Foot length and width, and footwear length and width was measured with a Brannock® device. Incorrectly fitted footwear was defined as 1 mm difference between length or width of the foot and footwear. Each participant completed FHSQ (Spanish version).	• Participants wearing incorrectly fitted shoes displayed lower FHSQ scores for section related to foot health and health status in general • Significant difference between the incorrect and correct footwear fitting groups for the dimensions of the FHSQ assessing pain, foot function, general foot health and social function
McHenry et al. [30]	56 participants, 45 men, 11 women, mean age 33.6 ± 11.7) yrs., height 174.9 ± 8.6 cm, weight 76.6 ± 12.5 kg. All participants were rock climbers with over 1 year of experience. Mean age of climbing experience 10.8 ± 11.2 yrs.	Foot length in bipedal stance was taken with ‘Ritz stick’. Climbing footwear was measured along its longest axis from the most posterior point of the heel to the furthest point anteriorly. For footwear with a downturned forefoot, shoe were flattened along the medial longitudinal arch. Incorrectly fitted footwear was defined as difference between foot and footwear greater or less 1 UK shoe size or equivalent.	• 55 (98%) participants were wearing excessively tight climbing footwear (based on length of foot and shoe) • Mean size reduction of 4 UK shoe sizes between participants street footwear and climbing footwear. • 51 (91%) participants experienced foot pain while climbing • 43 (76.8%) participants removed their footwear intermittently throughout activity to relieve discomfort
McInnes et al. [15]	203 participants, 85 participants with diabetes, 118 control participants without diabetes.	Both feet were measured using a Brannock® device during relaxed standing. Footwear dimensions were measured using a calibrated internal shoe size gauge. Incorrectly fitted footwear were defined as a difference between foot length and shoe size less than 10 mm or greater than 15 mm.	• 78 (66%) of participants were wearing footwear that were the incorrect size • 42 (55%) of participants were wearing footwear that were too short • 36 (47%) of participants were wearing footwear that were too long • In participants with diabetes, 70 people (82%) were wearing footwear that were the incorrect size • 30 (43%) were wearing footwear that were too short • 40 (57%) were wearing footwear that were too long
Menz and Morris [20]	176 participants, 56 men, 120 women, mean age: 80.1 ± 6.42 yrs. Participants were residing in retirement villages.	A footprint was taken relaxed weightbearing, The maximum length and width and area of the participant’s foot was measured. The outline of each shoe was traced onto graph paper, Fit of most regularly worn footwear was assessed. The percentage difference between the foot and footwear dimensions was calculated for length and width measurements.	• 23 participants (13.7%) wore indoor footwear shorter than their feet • 136 (81.4%) participants wore indoor footwear narrower than their feet • 73 (43.7%) wore indoor footwear smaller than the total area of their feet • 17 (10.2%) participants wore outdoor footwear shorter than their feet. • 131 (78.4%) participants wore outdoor footwear narrower than their feet • 79 (47.3%) participants wore outdoor footwear smaller than the overall area of the foot • Women displayed a greater disparity between foot and shoe dimensions with respect to indoor shoe length, indoor shoe width, indoor shoe area, outdoor shoe length, outdoor shoe width and outdoor shoe area • The presence of corns and callus was associated with inadequate footwear width • Moderate to severe hallux valgus was associated with inadequate width of indoor shoes and inadequate width and overall area of outdoor shoes • Lesser toe deformity was associated with inadequate length of both indoor and outdoor shoes. • Foot pain was associated with inadequate width of indoor shoes
Nixon et al. [25]	440 participants. 414 men, 26 women, mean age: 67.2 ± 12.5 yrs. All participants were war veterans recruited from veterans affairs medical centre. 58.4% of participants were diagnosed with diabetes and 6.8% had active diabetic ulceration.	Foot size was and width were measured during standing with using a standardised method and the Apex 1141 ft measuring device (Ritz stick). Incorrect sized footwear was defined as a size that was at least one full US shoe size too large or too small. The foot was also inspected for the presence of diabetic foot ulceration and peripheral neuropathy (protective sensation).	• 25.5% of participants were wearing appropriately sized footwear (based on length of foot and shoe) • Participants with diabetic foot ulceration were 5.1 times more likely to be wearing incorrectly fitted footwear than participants without a wound • Participants with diabetes and loss of protective sensation were 4.8 times more likes to be wearing incorrectly fitted footwear compared to participants without neuropathy
Schwarzkopf et al. [31]	235 participants. 71 participants from a private clinic, 25 male, 46 female mean age 45.2 yrs. 40 participants from a diabetes foot clinic, 18 male, 22 female, mean age 55.6 yrs. 124 participants from a charity care centre for the homeless, 124 male, 0 female, mean age 44.2 yrs.	Two foot and ankle surgeons measured foot length while standing using a length-measuring device (Clarks meter), foot length was represented as assumed US adult shoe sizes. Size of the participant current footwear was recorded. Incorrectly fitted footwear was defined as a difference of at least 0.5 US shoe sizes between measured foot size and the participants assumed footwear size.	• All participants: 82 (34.9%) were wearing incorrectly fitted footwear (based on length of foot and shoe) • 11 (15.5%) of participants from private clinic were wearing incorrectly fitted footwear • 17 (42.5%) of participants from diabetic foot clinic were wearing incorrectly fitted footwear • 54 (43.5%) participants from clinic caring for the homeless were wearing incorrectly fitted footwear • There were significant differences (*P* < 0.01) between the number of participants wearing incorrectly fitted footwear from the private clinic compared to participants from both the diabetic foot clinic and clinic for the homeless • 28 (11.9%) participants from all clinics were wearing incorrectly fitted footwear by greater than 1.5 sizes • 3 (4.2%) participants from private clinic were wearing incorrectly fitted footwear by greater than 1.5 sizes • 4 (10.0%) participants from diabetic foot clinic were wearing incorrectly fitted footwear greater than 1.5 sizes • 28 (16.9%) of participants from clinic for the homeless were wearing incorrectly fitted footwear greater than 1.5 sizes • There were significant differences (*p* < 0.01) between the number of participants wearing incorrectly fitted footwear greater than 1.5 sizes from the private clinic compared to participants from clinic for the homeless • Female gender was associated with shoe size mismatch (*p* = 0.02).

For all included studies, sample sizes ranged from 50 to 440 participants, with the median number of participants being 138. Mean or average age was reported in all but one study [15]. One study recruited only children [16] and four studies recruited only older participants [17–20]. Three studies recruited only female participants [21–23], while two other studies recruited predominantly male populations, including underground coal miners [24] and war veterans [25]. Participants with specific medical conditions were recruited in seven studies [15, 16, 25–29], including diabetes [25, 28, 29], diabetic peripheral neuropathy [15], Down’s syndrome [16], Alzheimer’s disease [27] and inflammatory arthritis [26]. Specific ethnic groups were examined in three studies including participants of Japanese [21], Thai [19] or Singaporean [26] ethnicity. Finally, four studies recruited specific populations [24, 25, 30, 31], including war veterans [25], underground coal miners [24], rock climbers [30] and three different population groups from New York City (a foot specialist private practice, an academic diabetic foot and ankle clinic, and a charity centre serving homeless people) [31].

### Study designs

The majority of included studies were cross-sectional in design [16–30] and three studies were case-control in design [15, 31, 32]. All except one study reported the method for measuring the foot [32]. The dimensions of the foot were measured during relaxed bipedal stance in all except two studies that measured the foot while the participant was in a seated position [18, 19]. It has been suggested that feet should be measured during standing to account for changes in dimensions due to splaying of the foot after the acceptance of bodyweight [33]. The studies that measured the foot while sitting did not provide a justification for doing so.

All except three studies measured the length of the foot, with most using a manual device, including a measurement stick [18, 25, 29–31], Brannock® device [15, 17, 26, 27] or callipers [19, 21, 28]. Two other studies analysed the area of a foot tracing [16, 20], while another measured the dimensions of a 3-dimensional scan taken from a foot mould [24].

Two studies only measured foot width [22, 23] while 10 studies measured foot width in addition to foot length [16–20, 24, 26–29], using a variety of manual devices such as a tape measure [19], Brannock® device [17, 26, 27] or callipers [18, 28, 29]. Four studies measured foot width using measurements from foot tracings [16, 20, 22, 23] while one analysed a 3-dimensional scan of a foot mould [24].

All except one study measured the shoes brought by participants to the testing session. The only study that did not measure the participants’ shoes asked participants to report their usual shoe size [28]. For all studies, footwear dimensions were measured using the same method as the measurement of the foot. Most studies used a manual device to measure footwear dimensions [15, 17–19, 21–23, 25–31]; two used measurements from a tracing of footwear [16, 20], and one study analysed a 3-dimensional mould of the internal dimensions of the shoe [24].

There were differences in the way that foot and shoe dimensions were compared and footwear fitting assessed. Several studies used a pragmatic approach guided by shoe sizing [15, 18, 19, 25–29, 31, 32]. For example, one study considered a shoe to be incorrectly fitted if it was at least half a British shoe size larger or smaller than the foot [31]. In contrast, other studies considered shoe fitting to be incorrect if measurements such as overall area, length or width differed between footwear and the foot [16, 20–24, 30]. In these cases, even though footwear and the foot may be different dimensions, footwear size may be correct. This is particularly relevant for length, as it has been suggested that appropriately sized footwear should have a space of at least 10–20 mm between the end of the foot and the shoe [33, 34]. In presenting results related to particular participant groups in this review, a distinction will be made between studies that deemed shoes to be incorrectly fitted based on shoe sizing, and studies that measured differences between the dimensions of the foot and the shoe.

### Prevalence of incorrectly fitted footwear

Most studies explicitly reported fitting variables such as length or width, or commented on overall footwear fit by reporting only one variable, usually length. However, there were five studies that reported the number of participants that were wearing incorrectly fitted shoes based on more than one variable [15, 18, 26, 27, 29]. Four studies reported that between 63 and 72% of participants were wearing incorrectly fitted shoes based on length and width [18, 29], while one study reported that 68% of participants wore incorrectly fitted shoes based on three measures (length, width and depth) [26]. One study compared total foot and shoe area to determine footwear fit, finding that the total area of the footwear was smaller than total area of the foot in 47% of participants [20].

Among the studies that provided specific fitting details, the percentage of participants wearing footwear too long for the foot ranged between 14 [32] and 73% [21] (median 38% [27]) and too short between 0.6 [28] and 98% [30] (median 10% [20]). In terms of width, between 30 [32] and 88% [23] (median 58% [27]) of participants wore footwear that were too narrow and one study found that only 1% of participants wore footwear that was too wide [28]. One study examined depth, finding that 31% of participants wore footwear that was too shallow [26].

### Association between footwear fitting, foot pain and foot disorders

The association between incorrectly fitted footwear and foot pain or foot disorders was investigated in eight studies [17, 18, 20, 23, 25, 28, 30, 31], with all but one study [31] reporting significant associations between incorrectly fitted footwear and some form of foot pain or foot disorder.

There were five studies that reported an association between incorrectly fitted footwear and foot pain or impaired quality of life [17, 18, 23, 28, 30]. These studies reported a strong association between tight footwear and foot pain, with between 84 [23] and 91% [30] of participants reporting generalised foot pain while shod. However, the characteristics of recruited participants influenced findings. For example, the study that found that 91% of participants described foot pain was conducted on rock climbers, who wear tightly fitted footwear to optimise contact with the climbing wall. There was also evidence that loose footwear was associated with foot pain, with 64% of participants reporting generalised foot pain while shod [23]. Regarding quality of life measures, one study that recruited 65 older people in Spain found those with ill-fitting shoes displayed significantly poorer overall foot health using the Foot Health Status Questionnaire [17]. In terms of specific regions of the lower extremity, one study reported that incorrectly fitted footwear was significantly associated with the presence of pain in the ankle in women [28].

There were three studies that investigated the association between incorrectly fitted footwear and foot disorders [18, 20, 25]. Among these there was evidence that incorrectly fitted footwear was associated lesser toe deformity in older people [20] and the presence of corns in older people [18]. Importantly, there was also evidence of a strong association between current foot ulceration and incorrectly fitted shoes in older people with diabetes [18, 25] with participants with current foot ulceration up to 5 times more likely to be wearing incorrectly fitted shoes compared to individuals without foot ulceration [25].

### Footwear fitting in specific populations

#### Footwear in children

Children’s feet are different to adult feet, both in shape and posture, and are constantly changing as the child grows [35]. In addition, the morphology of children’s feet are more malleable than adult feet [36]. Indeed, footwear has been used to correct pathological skeletal alignment and foot posture among children [37]. Therefore, correct shoe fitting in children is of paramount importance.

Only one article addressed footwear fitting in children (under 18 years old). The study focused on children with Down’s syndrome, who are known to have flatter, shorter feet and are more likely to have hallux valgus compared to children without Down’s syndrome [38, 39]. This study recruited 50 children with all genetic variants of Down’s syndrome (mean age: 10.6, SD 3.9, range 5–18 years) and found that 58% wore footwear that was too narrow for their feet [16]. This indicates that children with Down’s syndrome may be unable to acquire footwear that can accommodate the wider dimensions of the foot associated with the condition.

#### Footwear in adults

Five studies investigated healthy adults [21–23, 31, 32], with three recruiting young female participants [21–23]. Only one of the studies that recruited young females assessed footwear length. This study of young Japanese females found that in a sample of 51 students (average age 21 years), 75% were wearing footwear that was longer than their feet [21]. However, the maximum difference was 14 mm, which falls in the range for correctly fitted shoes based on the recommendation of 10–20 mm clearance between the foot and the shoe [33]. In contrast, 22% wore footwear that was shorter than their feet, thus indicating a potential issue with wearing footwear of inadequate length [21].

The two other studies that recruited young females only considered footwear width, specifically, width in the forefoot [22, 23]. The first study of 356 American women (average age 42, range 20 to 60 years) found that 88% were wearing footwear that was narrower than their feet, with the average discrepancy being 1.2 cm [23]. Among these women, 37% were wearing high heeled shoes, 49% wore flats and 14% wore sneakers. This was followed by a subsequent study by the same authors, using the same methods, that recruited 255 American women (average age 41, range 20–60 years) and found 86% of participants were wearing footwear that was too narrow, with an average discrepancy of 0.88 cm [22]. No information about shoe type was provided in this study. Both studies also reported that those with foot deformities such as hallux valgus displayed a greater discrepancy between footwear and foot width, however no information was provided as to whether this difference was significant [22, 23].

Two further studies recruited both female and male participants using convenience sampling of individuals that regularly attended foot and ankle clinics [31, 32]. These studies recruited participants of similar age (mean ages 44 and 49 years, respectively) and found that between 35 and 56% of individuals were wearing footwear of incorrect length [31, 32]. Only the study by Akhtar et al. [32] investigated width, and found that 64% of people were wearing footwear that was narrower than their feet.

#### Footwear in older people

There is evidence to suggest that the feet of older people are broader in the forefoot region and have a flatter medial longitudinal arch compared young people [40, 41]. Furthermore, older individuals with foot pain display a greater number of foot deformities including hallux valgus and clawed lesser toes compared to older people without pain [42]. These differences in foot morphology may pose a problem for fitting, as shoes are designed to have smooth contours, and are not designed to accommodate irregular bony shapes associated with foot deformity [43].

Three studies assessed footwear fitting in older people, with mean ages ranging between 69 and 80 years [18–20]. The literature indicated that footwear width was of particular concern in older people. For example, a study by Menz et al. of 176 older people (mean age 80.1, SD: 6.4, range 62–96 years) found that 78% wore outdoor shoes narrower than their feet [20]. Similarly, a study by Chaiwanichsiri et al. of 213 older Thai people (mean age 68.7, SD 5.4 years) found that 50% of women and 34% of men wore shoes that were too narrow [19]. In both cases, investigators compared width measurements between the widest region of the shoe and the forefoot. However, Chaiwanichsiri et al. [19] used a higher threshold to define an incorrectly fitted shoe (at least 5 mm difference between the foot and shoe) compared to Menz et al. who reported incorrect fitting if any discrepancy between shoe and foot width was recorded [20].

Burns et al. [18] provided additional context in relation to shoe width, finding that 33% of older individuals wore footwear that was the same width as the foot, but was too long, while 15% of individuals wore shoes that were simultaneously too wide and too long. This may indicate that, in order to accommodate a wider forefoot, some older people may be choosing shoes that are too long for their feet [18].

The majority of studies including older people have assessed the fit of shoes commonly worn outdoors [18–20]. However, the fitting of indoor shoes should not be overlooked, such is the relationship between indoor shoes and falls in older people [44]. As was the case with outdoor shoes, indoor shoes are commonly too narrow for the foot. This was confirmed by Menz et al., which found that among a sample of 176 older people, 81% of participants wore indoor footwear that was narrower than their feet [20]. Therefore, along with outdoor footwear it is apparent that indoor footwear is inadequately designed to accommodate the foot width of older people [44, 45].

The literature has suggested two reasons to explain the overall shoe fitting findings related to older people. Firstly, older individuals may be unable to select footwear to accommodate changes in foot morphology. For example, it may be difficult to acquire footwear that can accommodate a wider forefoot while also being appropriately fitted according to foot length [46]. Secondly, older individuals may not be aware of their correct shoe size. Indeed, a survey of 128 older people found that 26% of men and 47% of women had not had footwear size measured over the past 5 years [47]. If this is correct, then it is vital that health professionals ensure that all older patients are aware of their foot dimensions and appropriate footwear size for length and width.

#### Footwear in people with diabetes

The association between ill-fitting shoes and the development of foot ulceration in people with diabetes is well documented. Prospective studies have found that either ill-fitting footwear alone [48, 49], or minor trauma caused by footwear is the most common precipitating factor in the development of diabetic foot ulcers [50, 51].

Five studies have investigated shoe fitting among participants diagnosed with diabetes, with all using a similar approach to fitting analysis based on footwear sizing [15, 25, 28, 29, 31]. For example, two studies considered footwear fitting to be incorrect if there was half a size difference between the foot and footwear [29, 31] while one study applied a full size benchmark [25]. Other studies deemed footwear size inappropriate if the difference between foot and footwear length was outside a range of 10–15 mm [15] or greater than 2 mm [28]. All studies recruited similar samples of individuals with diabetes in terms of age (mean age range 55.6 to 67.2 years) however there was a large range of sample sizes (range 43 to 440). Among these studies, between 33 [29] and 82% [15] of individuals with diabetes were wearing shoes of incorrect length. Of these, between 10 [29] and 43% [15] were wearing footwear that was too short, while between 23 [29] and 81% (diabetic men only) [28] wore footwear that was too long.

In terms of shoe width, there was evidence among a sample of 568 diabetic individuals with peripheral neuropathy that the forefoot of most individuals with diabetes is broader than the most common industrial shoe width references used by shoe manufacturers [8]. This was supported by included literature in this review. For example, in a sample of 100 individuals (mean age 62.0 SD 14.9, range 24–89 years), 46% of individuals wore footwear that was too narrow [29]. However, of these participants, 67% wore the correct length footwear, which may indicate that a large proportion of individuals with diabetes may be selecting footwear that is correctly fitted for length, but are not considering, or are not able to acquire, footwear of sufficient width [29].

There was no evidence that a greater proportion of participants with diabetes wear incorrectly fitted footwear (according to length) compared to matched controls without diabetes [15, 25]. However, there was evidence of an association between incorrectly fitted shoes and the presence of diabetes-related foot lesions. For example, among a cohort of 440 male veterans with diabetes, those with current foot ulceration were 5 times more likely to be wearing incorrectly fitted shoes (at least one full shoe size difference) [25].

The overall findings suggest that, even though a similar proportion of individuals with and without diabetes wear incorrectly fitting footwear, the consequences of doing so for an individual with diabetes may be greater due to the potential development of diabetic foot ulceration.

#### Activity- or occupation-specific footwear

Footwear is often designed to fulfil a range of activity or occupational requirements. For instance, shoes may need protective or traction properties in order to allow the individual to safely and effectively carry out a required task [52]. One such is occupation is mining, which requires workers to stand for long periods of time on sometimes uneven, wet or unstable surfaces [53]. It was identified in a study of 208 mining workers that the boots worn by miners (both lace-up boots and gum boots) were significantly narrower than the foot but also significantly longer than the recommended 10–20 mm clearance [24]. These findings could indicate an attempt by miners to select shoes of appropriate width by wearing excessively long shoes. This in turn may contribute to the high prevalence of foot pain reported in this population [53].

The only study that investigated activity specific footwear indicated that, among 56 rock climbers, all participants wore shoes that were smaller than the foot, with the mean difference being the equivalent of four British shoe sizes [30]. However, despite the reported pain, these shoe-fitting habits were deemed necessary by rock climbing participants to attain enhanced performance by ensuring close contact between the foot and the climbing surface.

#### Footwear fitting differences between sexes

Women are more likely to suffer foot pain compared to men [54, 55]. Furthermore, females suffer more foot pain while wearing shoes compared to men, most likely due to the narrower toe box of women’s shoes [56]. Of the studies that recruited both women and men, only one compared shoe fitting between sexes. In this study of older individuals, percentage difference between measures of length, width and total area was compared for both outdoor and indoor shoes [20]. In all comparisons, women displayed significantly greater percentage differences compared to men, thus indicating that women were wearing relatively smaller footwear.

Three studies investigated footwear fitting among men and women, but did not carry out significance testing to compare fitting between the two sexes [19, 28, 31]. These studies provide some evidence that a greater proportion of women wear incorrect sized footwear compared to men. For example, one study reported a greater proportion of women wore shoes that were too narrow (difference greater than 5 mm between foot and shoe) compared to men (50% women, 34% men) [19]. However, there is also some evidence that a greater proportion of men were wearing footwear that was too long compared to women. This was evident in a study of older individuals with diabetes, including 227 women and 172 men that found a greater percentage of men were wearing incorrectly fitted shoes (69% men, 48% women) [28]. Of these 31% of men and 13% of women were wearing shoes that were at least 10 mm longer than their feet.

## Conclusions

The available evidence indicates that a large proportion of the population (between 63 and 72%) are wearing inappropriately sized footwear based on length and width measurements, and that incorrect footwear fitting is significantly associated with foot pain, poorer overall foot health, corns and calluses in older people and foot ulceration in older people with diabetes. However, a limitation of the literature is that there are variations between studies in the way that footwear fitting is measured and defined. Hence, future work should adopt standardised approaches to assessing footwear fitting.

Of particular interest among the included literature are findings related to specific groups of participants that are more likely to display variations in foot morphology compared to the broader population. These participants include children with Down’s syndrome, older people, or people with diabetes. Fitting according to foot width was a particular concern among these groups with between 46 and 81% of participants wearing footwear that was too narrow. In addition, there is evidence that older people and people with diabetes may select footwear of inappropriate length in order to acquire footwear to accommodate forefoot width.

Overall, the high prevalence of incorrectly fitted footwear suggests that greater emphasis should be placed on footwear fitting education so people are more aware of their foot dimensions and appropriate foot size. Furthermore, footwear manufacturers should provide an appropriately large selection of shoes that can accommodate the variations in foot morphology among the population. In particular, a greater range of widths for each length sizing option should be made available in order to accommodate feet with wider dimensions.
